# Ongoing involvers and promising therapeutic targets of hepatic fibrosis: The hepatic immune microenvironment

**DOI:** 10.3389/fimmu.2023.1131588

**Published:** 2023-02-16

**Authors:** Nana Zhang, Huimin Yao, Zhixuan Zhang, Zhuoqun Li, Xue Chen, Yan Zhao, Ran Ju, Jiayi He, Heli Pan, Xiaoli Liu, Yi Lv

**Affiliations:** ^1^ Institute of Regenerative and Reconstructive Medicine, Med-X Institute, First Affiliated Hospital of Xi’an Jiaotong University, Xi’an, China; ^2^ National Local Joint Engineering Research Center for Precision Surgery and Regenerative Medicine, First Affiliated Hospital of Xi’an Jiaotong University, Xi’an, China; ^3^ Shaanxi Provincial Center for Regenerative Medicine and Surgical Engineering, First Affiliated Hospital of Xi’an Jiaotong University, Xi’an, China; ^4^ Department of Hepatobiliary Surgery, First Affiliated Hospital of Xi’an Jiaotong University, Xi’an, China

**Keywords:** hepatic fibrosis, hepatic immune microenvironment, chronic liver diseases, hepatic stellate cells (HSCs), immune cells

## Abstract

Hepatic fibrosis is often secondary to chronic inflammatory liver injury. During the development of hepatic fibrosis, the damaged hepatocytes and activated hepatic stellate cells (HSCs) caused by the pathogenic injury could secrete a variety of cytokines and chemokines, which will chemotactic innate and adaptive immune cells of liver tissue and peripheral circulation infiltrating into the injury site, mediating the immune response against injury and promoting tissue reparation. However, the continuous release of persistent injurious stimulus-induced inflammatory cytokines will promote HSCs-mediated fibrous tissue hyperproliferation and excessive repair, which will cause hepatic fibrosis development and progression to cirrhosis even liver cancer. And the activated HSCs can secrete various cytokines and chemokines, which directly interact with immune cells and actively participate in liver disease progression. Therefore, analyzing the changes in local immune homeostasis caused by immune response under different pathological states will greatly enrich our understanding of liver diseases’ reversal, chronicity, progression, and even deterioration of liver cancer. In this review, we summarized the critical components of the hepatic immune microenvironment (HIME), different sub-type immune cells, and their released cytokines, according to their effect on the development of progression of hepatic fibrosis. And we also reviewed and analyzed the specific changes and the related mechanisms of the immune microenvironment in different chronic liver diseases.Moreover, we retrospectively analyzed whether the progression of hepatic fibrosis could be alleviated by modulating the HIME.We aimed to elucidate the pathogenesis of hepatic fibrosis and provide the possibility for exploring the therapeutic targets for hepatic fibrosis.

## Introduction

Hepatic fibrosis is a reversible wound-healing response to liver injury, which is a major feature of any form of the chronic liver disease progressing to cirrhosis and liver failure (1). The main manifestations of hepatic fibrosis are chronic liver injury and accumulation of extracellular matrix (ECM) proteins (2). The continuous accumulation of ECM proteins could disrupt the normal function of the liver, and persistent hepatic fibrosis will progress to liver cirrhosis and even hepatocellular carcinoma (3). However, no effective drugs have been approved to target hepatic fibrosis. The therapeutic approaches to alleviate hepatic fibrosis mainly include: controlling or removing the underlying causes of hepatic fibrosis, preventing the activation and proliferation of HSCs, inhibiting the overexpression of pro-fibrotic cytokine TGF-β, and promoting fibrous tissue degradation. An emerging stem cell therapy is that the stem cells can transform into functionally active hepatocyte-like cells and participate in liver function repair and reconstruction (4).

It has been confirmed that hepatic fibrosis is driven by the activation of HSCs (5), which is the main source of ECM (6). HSCs are located in the perisinusoidal space and maintain a non-proliferative and quiescent phenotype in the normal liver tissue. The HSCs will transdifferentiate into proliferative fibrotic myofibroblasts, acquiring pro-inflammatory and pro-fibrotic properties under the chronic injury caused by many various pathogenic factors such as pathogenic microorganisms, alcohol, chemical drugs, and lipid deposition (7). The myofibroblasts transformed from HSCs account for about 82%-96% of myofibroblasts in various chronic liver diseases (8). Therefore, activated HSCs are often regarded as the main therapeutic target.

HIME is a dynamic network system composed of a variety of immune cells, immune cytokines, and related ECM (9), and it closely affects the onset and progression of hepatic fibrosis. Various immune cells are enriched in liver tissue and involved in the physiological and pathological process of hepatic metabolism and disease (10). Immune cells in the liver are diverse at a stable state and evolve further during the development of chronic liver disease, directly affecting the severity of the disease (11, 12). The recruitment and activation of immune cells in the diseased liver tissue are often initiated by the massive release and activation of cytokines and chemokines. Hepatic kuffer cells (KCs) are activated by various damage-associated molecular patterns (DAMPs, such as free DNA, ATP, high mobility group box 1) and pathogen-associated molecular patterns (such as LPS or viral DNA) that interact with Toll-like receptors (TLRs) or recombinant purinergic receptor P2X, ligand-gated ion channel 7 when hepatocytes are damaged (13, 14). This leads to the activation of the inflammasome, and the release of interleukin-1β (IL-1β), IL-18, and various other pro-inflammatory cytokines as well as chemokines. It will promote the recruitment of circulating leukocytes (monocytes and neutrophils), and activate adaptive immune T cells function. Once the infection or injury is controlled, the KCs and macrophages transform into anti-inflammatory and tissue repair phenotypes to control excessive tissue-damaging inflammatory responses (15). However, the persistent injurious stimulus in the liver will result in inflammatory cytokines continuously releasing that promote HSCs-mediated fibrous tissue hyperproliferation and excessive repair, which causes hepatic fibrosis development and progression to cirrhosis (16).

Hepatic fibrosis is an intermediate link and reversible stage in the process of chronic liver disease developing into liver cirrhosis. Intervention in this link will determine the prognostic direction of the various liver diseases. However, there is no efficient treatment strategy for hepatic fibrosis, which is mainly because profound awareness of the changes in the microenvironment of hepatic fibrosis still lacks, and no targeting and efficient intervention on the hepatic fibrosis microenvironment. Exploring the changes in local immune homeostasis and microenvironment caused by immune response under this pathological state could greatly enrich our understanding of liver disease reversal, chronicity, and even deterioration to liver sclerosis. Therefore, it is of great significance to elucidate the HIME for the study of hepatic fibrosis and the development of therapeutic strategies. In this review, we first summarized the critical components of HIME, the alteration and function of different sub-type immune cells, and their related mechanisms according to their effect on the development of progression of hepatic fibrosis. The specific changes and the related mechanisms of HIME in different chronic liver diseases were also reviewed and analyzed. Finally, we also summarized the therapeutic approaches and targets for HIME to reverse the progression of hepatic fibrosis, and we aimed to provide new research ideas and the possibility of exploring the therapy for hepatic fibrosis.

## Influence of the immune cells on the development of hepatic fibrosis

The immune cells are central members of the immune microenvironment in the progression of hepatic fibrosis, and they can mediate the immune response by synthesizing and secreting different chemical inflammatory mediators. The immune cells affect the function and number of HSCs mainly through direct or indirect effects, thus influencing the progression of hepatic fibrosis. Simultaneously, they constitute a dynamically evolving microenvironment and interact as well as restrict each other, forming a complex network system, which jointly affects the process of hepatic fibrosis.

## Monocytes/macrophages

Hepatic macrophages include liver-resident KCs and monocyte-derived macrophages (MDMs) (17). The MDMs, with high heterogeneity and plasticity, can differentiate into functionally distinct macrophage subsets. In mice, the circulating macrophages can be divided into the Ly6C^hi^ CCR2^hi^ CX3CR1^lo^ monocytes which are considered to be potent pro-inflammatory cells, and Ly6C^lo^ CCR2^lo^ CX3CR1^hi^ monocytes, which with an anti-inflammatory patrolling function (18). Ly6C^hi^ monocytes in response to liver chronic injury are recruited to the liver and differentiate into MDM. These Ly6C^hi^ MDMs have been shown to promote liver fibrosis, and they could promote HSCs activation as well as proliferation and ECM production by expressing a large number of pro-fibrotic mediators. The MDMs are also capable of differentiating into Ly6C^lo^ restorative macrophage phenotypes, which promote ECM degradation and fibrosis resolution (19). KCs, the settled macrophages in the liver, have been identified as key regulators of hepatic inflammation. The DAMPs released from damaged hepatocytes can activate KCs and promote circulating macrophage infiltration in the liver (12). The activated macrophages have been reported to possess a bidirectional effect on the onset, progression, and reversal of hepatic fibrosis (17). Activated KCs could activate the HSCs by secreting large amounts of pro-inflammatory factors such as TGF-β1, IL-1β, and tumor necrosis factor-α (TNF-α) as well as the chemokines (20, 21). The TGF-β1 and platelet-derived growth factor (PDGF) are the most important inflammatory mediators in hepatic fibrosis. TGF-β1 regulates the activation of HSCs through the Smad pathway and inhibits the degradation of the ECM (22). PDGF activates HSCs and promotes their proliferation through the subsequent phosphatidylinositol 3 kinase (PI3K) and extracellular signal-regulated kinase pathways (23). Additionally, macrophages also mediate the reversal of fibrosis. Firstly, macrophages secrete a series of matrix metallic-proteinases (MMPs), which specifically degrade collagen and non-collagen extracellular matrix (24). Secondly, macrophages mediate HSCs apoptosis by expressing TNF-related apoptosis-inducing ligand (25), leading to the reduction of ECM and alleviation of hepatic fibrosis (26). Relaxin is an anti-fibrotic peptide hormone that can directly reverse the activation of HSCs to promote the resolution of hepatic fibrosis (27). A study showed that hepatic macrophages express primary relaxin receptors and they switch from a profibrogenic to the pro-resolution phenotype upon binding with relaxin. The latter releases exosomes that promote relaxin-mediated quiescence of activated hepatic astrocytes *via* miR-30a-5p (28). This indicates that macrophages have great potential in anti-fibrosis research and can be used as a target for anti-fibrosis therapy or reversal of fibrosis.

## Dendritic cells

Dendritic cells (DCs) are the specialized antigen-presenting cells, with the function of transforming immune tolerance to immune activation, regulating the direction of the immune response in HIME of hepatic fibrosis (29). During hepatic fibrosis, DCs proliferate and undergo phenotypic changes. They stimulate adjacent T cells and natural killer(NK) cells through TNF-α, and up-regulate intercellular adhesion molecule-1 and CD40 expression of HSCs, thus, promoting the HSCs proliferation and activation (30, 31). DCs also aggravate liver inflammation by inhibiting infiltration of Treg cells and activating TLRs to produce a large number of cytokines (32). And the DCs depletion completely abrogates the elevated levels of many inflammatory mediators that are produced in the fibrotic liver (33). A similar study found knockout of DCs reduced the clearance of activated HSCs and delayed the fibrotic reversal process during the reversal phase of CCL4-induced hepatic fibrosis in mice (34).However, it also reported that although DCs can promote the activation of HSCs, the depletion of DCs does not affect the evolution of liver fibrosis in CCL4-induced hepatic fibrosis (35). Moreover, the application of FMS-liketyrosinekinase3 ligand induced the proliferation of DCs, accompanied by increased MMP-9 secretion from DCs. And the MMP-9 not only directly degrades collagen but also recruits innate immune cells such as macrophages and neutrophils, secreting MMP-8 and MMP-13 to degrade collagen, facilitating the reversal of hepatic fibrosis (34). Besides, long-term (12 weeks) alcohol intake can specifically recruit plasmacytoid dendritic cells to the liver in female mice. These plasmacytoid dendritic cells are characterized by secreting IFN-α and with anti-fibrosis function (36). These results suggest the effect of DCs in hepatic fibrosis is still controversial and need to be revealed and defined in the future research.

## Natural killer cells

NK cells in liver were subdivided into CD49a^+^DX5 ^–^ liver-resident natural killer (lrNK) cells and CD49a^-^ DX5^+^ conventional natural killer (cNK) cells. Both of them could kill activated HSCs in a tumor necrosis factor-related apoptosis-inducing ligand dependent manner (37). Compared to cNK cells, lrNK cells are less mature at a rest statue, but it have enhanced activity upon pathogenic stimuli and exhibit higher cytotoxicity (38). It suggested that the enrichment of lrNK cells in the liver may be to prepare for a special function against liver injury. It also was reported that lrNK cells can highly express CD107a and perforin, work together with a variety of activated receptors, release interferon-γ (INF-γ) while splitting infected hepatocytes, and inhibit the activation of HSCs in the human viral hepatitis (39). In the early stage of hepatic fibrosis, cNK cells infiltrate into the liver parenchyma and alleviate or reverse hepatic fibrosis by directly killing or inducing apoptosis of HSCs through degranulation and TNF-related apoptosis-inducing ligand expression (40). Moreover, the activated NK cells release IFN-γ through the JAK-STAT pathway to antagonize hepatic fibrosis by exerting a killing effect on the activated HSCs (41, 42). The activated NK cells can also secrete IL-10 and IL-22,which promote the senescence of activated HSCs and alleviate liver fibrosis through signal transducer-activator 3 (STAT3)/p53/p21 pathway (43, 44). And the activation of metatropic gluamate receptor 5 in NK cells can also reduce liver fibrosis by increasing their cytotoxicity and IFN-γ production (45).The NKp46(+)NK· cells were reported to attenuate metabolism-induced hepatic fibrosis by regulating macrophage activation in mice (46). In patients with chronic HVB infection, the terminally differentiated NK cells often highly express CD57 and DNAM-1, while NKp46 and NKG2A expression at low levels. These NK cells can kill activated HCSs through tumor necrosis factor-related apoptosis-inducing ligand (TRAIL) dependent and CD44-osteopontin dependent manners (47). In contrast, during the progressive phase of hepatic fibrosis, persistent activation of HSCs will inhibit NK cell activity and weaken its anti-fibrotic effect. It is attributed to the increased metabolism of vitamin A. Its metabolites, retinoic acid, and retinol can inhibit the activation of IFN-γ on the transcriptional STAT-1 pathway by secreting the suppressor of cytokine signaling 1 (SOCS1) (41, 48).

NKT cells, a subset of T cells, possess NK and T cell receptors and display both NK cell and T cell properties. Similar to the NK cells, NKT cells also inhibit hepatic fibrosis by directly killing the activated HSC cells or producing IFN-γ to exert the inhibiting effect on HSCs. In the CCL4-induced acute liver injury mouse model, the hepatic NKT can slow down the onset of acute liver injury and liver inflammation by inhibiting activated HSCs (49). However, the anti-fibrotic effect of NKT cells is limited to the acute liver injury period, and in persistent chronic liver injury, the anti-fibrotic effect of NKT is diminished due to functional failure of NKT and immune tolerance of the liver (50). Additionally, Park et al. found that α-galactosyl neurosphingosamine overactivated NKT cells will accelerate CCL4-induced acute liver injury, inflammation, and fibrosis (51). In the progression of the alcoholic liver, NKT cell activation can exacerbate liver injury and promote an inflammatory response (52). Another study found that NKT cells from viral-infected patients could secrete more pro-inflammatory factors IL-4 and IL-13 compared with those from non-infected patients. It suggests that NKT cells may promote fibrosis by producing pro-inflammatory factors IL-4 and IL-13 to promote the development of fibrosis in patients with chronic viral infection (53).

## T cells

Different subtypes of T cells have different effects on hepatic fibrosis. Th1 and Th2 cells play the core role in this process, and they tend to exhibit mutually antagonistic effects on hepatic fibrosis. IFN-γ secreted by Th1 cells regulates the balance of MMP and tissue inhibitor of metalloproteinase (TIMP), which can inhibit HSCs activation and proliferation, induce HSCs apoptosis, and inhibit TGF-β expression in a variety of cells (e.g., hepatocytes, KCs, HSCs) to exert anti-fibrotic effects (54, 55). IL-13 secreted by Th2 induces the production of collagen I, collagen III, α-smooth muscle actin, and TIMP-1 to induce TGF-β secretion, activate HSCs, and inhibit HSCs apoptosis thus promoting the progression of hepatic fibrosis (56). Moreover, IL-4, IL-5, and IL-13 secreted by Th2 cells reduce the liver inflammatory response, but also inhibit the Th1-mediated cellular immune response and prevent the clearance of viruses and parasites, leading to the persistence of viruses and inflammation (57). In addition, Th1 and Th2 cells regulate cellular collagen synthesis by antagonistically regulating nitric oxide synthase 2/arginase activity (58). In a non-alcoholic steatohepatitis (NASH) mouse model, mice lacking the IFN-γ (typical Th1 cytokine) showed significant protection against liver damage and fibrosis (59).

CD8^+^ T cells mainly produce cytotoxic molecules such as IFN-γ, TNF, and perforin (60). During NASH in mice, the number of CD8^+^ T cells in the liver was increased, and lipid-conditioned CXCR6^+^ CD8^+^ T cells induce hepatocyte killing in a perforin-independent, FasL (CD95L)-dependent manner (61). Consequently, in a diet-induced mouse model of NASH, CD8^+^ T cell depletion blunted liver injury (62). Thus, CD8^+^ T cells are likely to promote hepatic injury during NASH. But the mechanism by which T cell subsets are activated and interact to promote liver inflammation is not well understood. And more research is needed to investigate the specific mechanism and how to target these pathways without compromising immune defenses (63). Additionally, hepatic tissue-resident memory T cells (TRM cells) stably occupy tissues and participate in hepatic fibrosis progression differing from T cells in the circulation. CD8^+^T cells characterized by CD69 and CD103 are defined as CD8^+^ TRM cells (64, 65). Koda Y et al. found that CD8^+^ TRM cells were able to attract HSCs in a CCR5-dependent manner and mediated apoptosis of activated HSCs through the Fas- FasL pathway (66). Although little latest work had been done to verify the exist of CD4^+^TRM fraction in human liver, its relationship with liver fibrosis has not been systematically studied and documented (67).

Gamma-delta T cells(γδ T cells), as liver tissue-resident T cells, are characterized by the gamma and delta chains of T cell receptors (68, 69). Hepatic γδ T cells account approximately for 3% - 5% of the hepatic lymphocytes and 15% - 25% of the hepatic T cells, which are identified as liver-resident cells with predominant production of IL-17as well as the IFN-γ (70). And the IFN-γ will activate macrophages to release IL-15, prompting γδ T cells to accumulate at the infectious site and participate in local anti-inflammatory and anti-fibrotic effects, whereas IL-17, a major pro-inflammatory factor, often plays a pro-fibrotic effect on the fibrosis progress (70, 71). In the CCl4-induced hepatic fibrosis murine model, γδ T cells could activate the mTOC2 signaling pathway in response to macrophages, promoted CXCR3 transcription, and drove γδ T cell accumulation in the liver (72). It also documented that hepatic γδ T cells could kill the activated HSCs in a TRAIL-or FasL-dependent manner and secrete IFN-γ to inhibit differentiation of pro-fibrotic Th 17 cells to relieve hepatic fibrosis. And it also promoted HSC lysis by enhancing the cytotoxicity of conventional NK cells and liver-resident NK cells on activated HSCs (37). However, hepatocyte-derived exosomes mediated TLR3 activation in HSCs and increased IL-17 production from γδ T cells, and the IL-17 strongly stimulated the expression of a-SMA, TGF-β, IL-6, and collagen type I-α1 in HSCs and aggravated liver fibrosis (73).

## B cells

B cells are classified as B-1 cells, B-2 cells, effector B cells, and regulatory B cells (Bregs) based on their surface molecular, localization, and functional characteristics. Relatively few studies indicated that B cells can influence the development and progression of hepatic fibrosis in an antibody-independent manner (74). It has been demonstrated that B cells knockout mice given CCL4 injections had significantly lower hepatic fibrosis than normal control mice, suggesting that B cells have a pro-fibrotic effect and this effect is independent of antibodies and T cells (75). Conversely, B cells can also exacerbate hepatic fibrosis by indirectly affecting T lymphocyte function and contact with fibroblasts, phagocytes, and NK cells through the secretion of IL-1, IL-6, and IL-4 (76). Secondly, it has been found by retrospective analysis that the number of B lymphocytes was significantly lower in the cirrhotic group than in the hepatitis group and healthy individuals, which also implies that B lymphocytes are affected in the progression of cirrhosis and are involved in the development of cirrhosis (5). In addition, similarly to T cells, regulatory B cells (Bregs) also secrete cytokines inducing immune tolerance and maintaining environmental homeostasis. Bregs from patients with chronic hepatitis B or hepatitis B virus(HBV)-associated hepatic fibrosis can inhibit the function of Th1 and Th17 through intercellular contact and secretion of IL-10. Bregs can also induce the conversion of CD4^+^CD25^-^T cells to Tregs, resulting in inflammatory response suppression and inflammatory repair (77).

**Figure 1:**
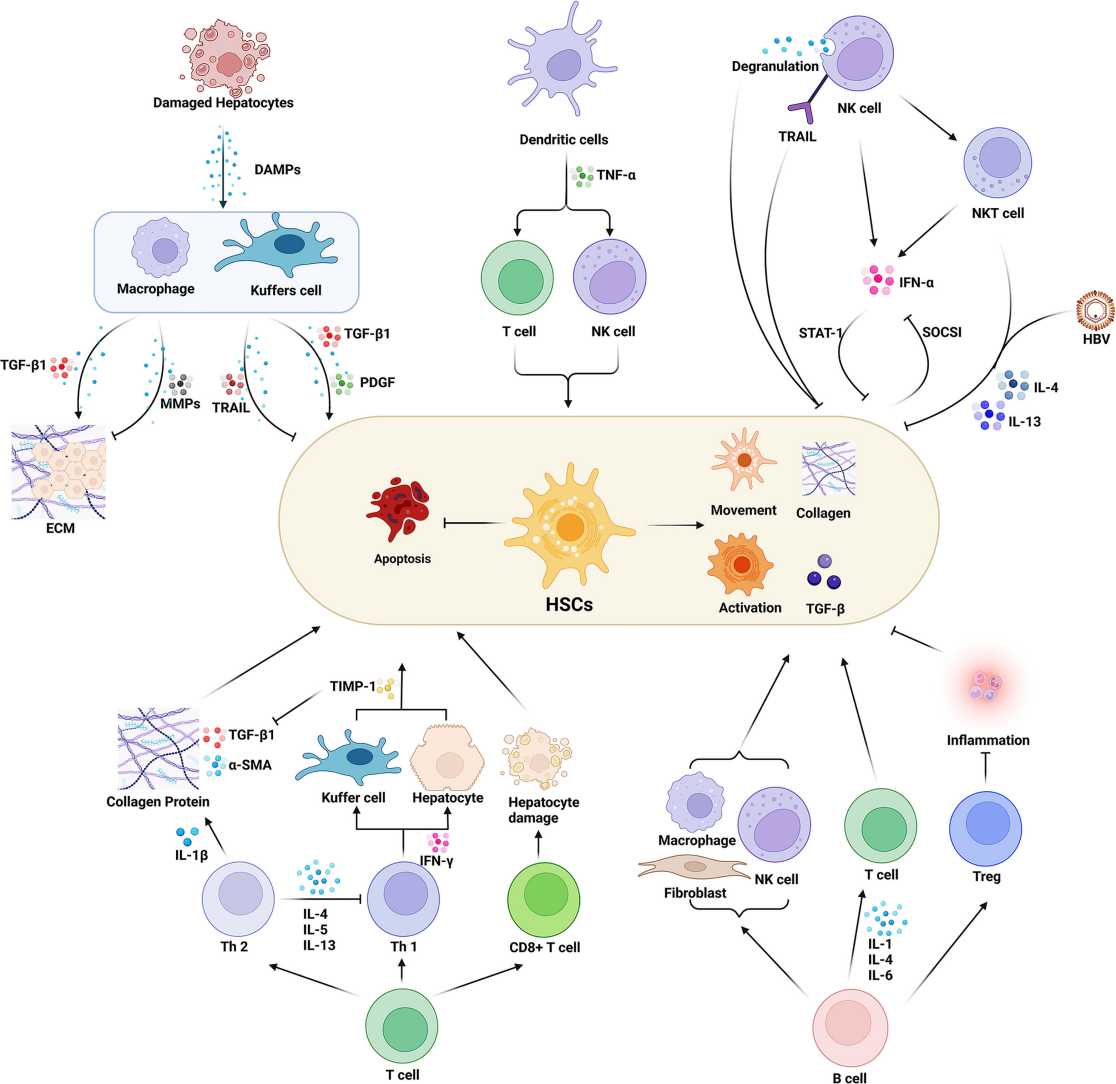
Effect of various immune cells in the hepatic immune microenvironment (HIME) on hepatic fibrosis. Various immune cells affect the process of hepatic fibrosis by promoting or inhibiting the function of HSCs. Macrophages play a dual role in this process. The peripheral macrophages and the resident liver macrophages (Kuffer cells) are activated under the damage-associated molecular pattern released from the damaged hepatocytes during injury or inflammatory conditions. Activated macrophages secrete proinflammatory factors such as TGF-β to promote ECM formation and activate HSCs. On the other hand, macrophages can also inhibit hepatic fibrosis by expressing MMPs and TRAIL. Dendritic cells secrete TNF-α to activate HSCs by enhancing the immune function of T cells and NK cells. And the activated NK cells inhibit the function of HSCs and prevent hepatic fibrosis progression. While NK cells also can directly damage HSCs through degranulation and up-regulation of TRAIL expression. Moreover, it can secrete INF-γ, and synergize NKT cells to inhibit HSCs activation through the STAT signaling pathway. However, HSCs can inhibit INF-γ secretion of NK cells *via* the SOCSI pathway. The balance of Th1/Th2 cells plays an important role in the process of hepatic fibrosis. Th1 cells secrete INF-γ to increase the expression of TIMP in macrophages and inhibit the activity of HSCs. Th2 cells can secrete a variety of cytokines to activate HSCs and inhibit the HSCs’ apoptosis as well as the activity of Th1 cells. CD8^+^ T cells secrete cytotoxic factors to mediate hepatocyte injury, thereby activating HSCs. B cells regulate macrophages, fibroblasts, and T cells and promote HSCs activation by secreting interleukins. And B cells are also able to regulate Treg cells to inhibit inflammatory responses and HSCs’ activation. Created with BioRender.com.

In summary, the immune cells directly act on HSCs through the interaction of ligands and receptors or by secreting cytokines to regulate the activity of HSCs. Additionally, immune cells play an immunomodulatory role or indirect effect on HSCs regulating the inflammatory response through the synthesis and secretion of different cytokines and chemokines, thus promoting or inhibiting the formation and progression of hepatic fibrosis. We depicted how the main sub-types of immune cells in the HIME influence the HSCs in **Figure 1**.

## The effect of HIME for different chronic liver diseases on the hepatic fibrosis

### The HIME of viral hepatitis

Viral hepatitis is a type of infectious disease caused by the hepatitis virus and characterized by hepatocyte degeneration, necrosis, and apoptosis. Chronic hepatitis, especially chronic hepatitis B, undergoes fibrosis with the virus-induced long-term inflammation and leading to the evolutionary pathway of “hepatitis–cirrhosis–liver cancer” (78, 79).

The major mechanisms of viral hepatitis progression to cirrhosis are the direct and indirect activation of HSCs (6). The Hepatitis virus can promote HSCs activation by disturbing immune cell functions, thereby exacerbating hepatic fibrosis (80). Xie et al. (81) found that the core antigen HBeAg of HBV is the most important component of HBV-induced macrophage activation. HBeAg binds with macrophages through the TLR-2 receptor, activates the NF-κB signaling pathway in macrophages, and releases a large number of cytokines and chemokines to inhibit viral proliferation and regulate HSCs’ function, producing different pro-fibrotic effects. On the one hand, TNF-α and IL-1β can inhibit HSCs apoptosis by up-regulating the expression of TIMP-1 and down-regulating the expression of BMP and activin membrane-bound inhibitor of HSCs (82–85). Macrophages are also able to up-regulate PI3K-AKT-mTOR and p38 mitogen-activated protein kinase (p38 MAPK) pathway in HSCs to promote the HSCs movement, and also activate TGF-β/smad pathway to promote their proliferation and contraction, and promote the production of type I and type III collagen (86). Moreover, the activated HSCs further affect the function of macrophages, and they interactively promote the progression of hepatic fibrosis. Akt/PKB (protein kinase B) in HSCs promotes fibrogenic M2 polarization in macrophages (87). HBeAg directly induces up-regulate the secretion of TGF-β of HSCs, and the secreted TGF-β positively feeds back to HSCs themselves and mediates their activation and proliferation (88). The HBV and its related proteins reduce the expression of TLR in macrophages, leading to HBV immune tolerance and persistent infection. The described mechanisms of macrophage and HSCs interacted in the liver for a long time, allowing hepatic fibrosis occurrence and progress to cirrhosis or even hepatocellular carcinoma (78, 89).

Viral infection could inhibit NK cells to produce the anti-fibrotic cytokine IFN-γ, promoting the development of hepatic fibrosis. A study found that HBV infection down-regulates the expression of NK cell activating receptors NKG2D and 2B4 on NK cells *via* TGF-β1 secreted by hepatocytes, resulting in a decreased expression of their intracellular adaptor proteins DAP10 and SAP, which impair NK cell-mediated cytotoxicity ability and IFN-γ production (90). Another study found that ability to the production of IFN-γ of CD56 (dim) NK cells in patients with chronic HBV infection is impaired, leading to an increased NKG2A expression and decreased CD16 expression, thereby preventing NK cells activation and promoting hepatic fibrosis (91). Activation of the PDGF/PDGF-β receptor also plays a critical role in hepatic fibrosis. HBV core protein and HBV regulatory X protein are two proteins expressed by HBV, which can promote PDGF production by increasing the transcription of PDGF in hepatocytes. PDGF will bind to the PDGF-β receptor, a receptor located on HSCs, induce receptor phosphorylation, and initiate various intracellular signaling pathways activation such as the JAK/STAT, PI3K, PLC-γ, or MAPK pathways, leading to HSC migration, proliferation, and ECM secretion (92–94).

**Figure 2:**
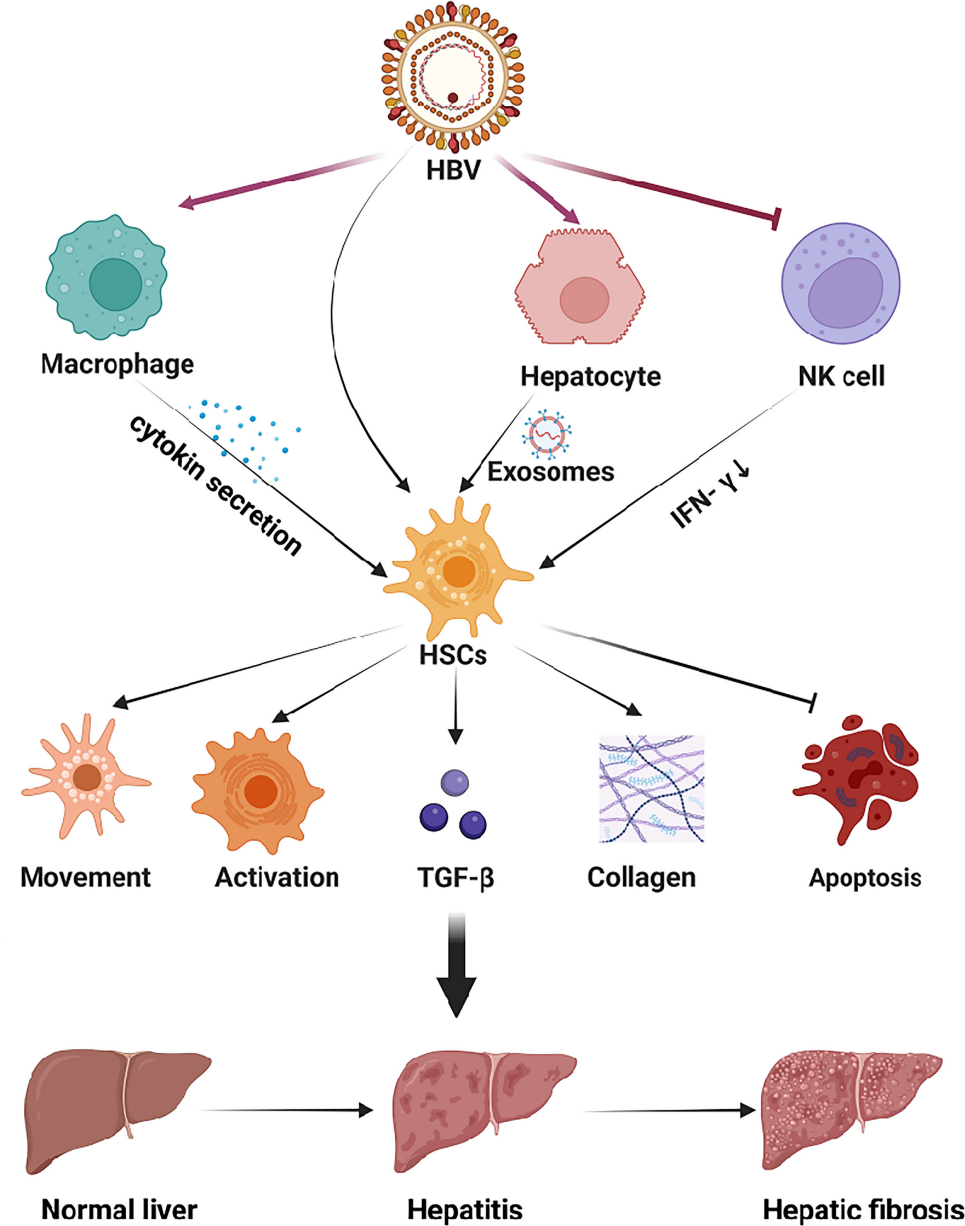
The HIME of HBV-induced viral hepatitis. The hepatitis virus replicates within hepatocytes and is secreted into the extracellular space *via* e exosomes. Viral antigens can exert multiple effects in HSCs to promote hepatic fibrosis. In addition, the virus can be recognized by macrophages and NK cells, which will up-regulate or down-regulate the secretion of cytokines to activate HSCs. Subsequently, HSCs activation aggravates hepatitis and promotes the progression of hepatic fibrosis. Created with BioRender.com.

In the virus-infected HIEM, exosomes secreted by hepatocytes, containing miRNAs, also can regulate the function of HSCs. MiR-19, an exosome secreted from HCV-infected hepatocytes, could reduce SOCS3 production of HSC. And the SOCS3 could block JAK kinase, thus the reduction of SOCS3 will activate the JAK-STAT3 pathway, increase cyclin D1 transcription, and stimulate HSC proliferation (95). HCV-infected hepatocytes also secrete miR-192-containing exosomes and exert an effect on HSCs, increase TGF-β1 expression, and stimulate HSCs activation and differentiation into myofibroblasts phenotype (96). **Figure 2** summarized the effect of viral hepatitis-induced HIME changes on the development and progression of hepatic fibrosis.

### The HIME of autoimmune liver diseases

Autoimmune liver diseases (AILD) are mainly characterized by liver injury along with elevated serum immunoglobulins and the presence of multiple auto-antibodies in the blood, which is associated with autoimmunity. It can be divided into autoimmune hepatitis, primary sclerosing cholangitis, and primary biliary cholangitis (PBC), according to the cell type involved (97).

As the autoimmune tolerance of patients with AILD is impaired, a number of auto-antibodies are produced and accumulated in the HIME, including antinuclear antibodies in autoimmune hepatitis, antimyosmooth muscle antibodies, and antimitochondrial antibodies in PBC. Driven by humoral immunity, the activated CD4^+^ T cells (including Th1 and Th2) stimulate B cells to produce antibodies against auto-antigens by directly T-B cell membranes contacting and releasing cytokines, and with chronic stimulation on hepatocytes. The damaged hepatocytes could recruit the NK cells and macrophage infiltration that exacerbate the inflammatory response within the liver (63, 98). Wu et al. (99) showed that the transcription of POU6F1 in biliary epithelial cells activates monocyte chemotactic protein-1 (MCP-1) and promotes peripheral M1 and M2 macrophage recruitment and infiltration into peribiliary gland (PBG) niche, producing chronic inflammation, resulting in dilated PBG compartments and the formation of “onionskin” fibrosis characterized by multifocal fibrosis around intrahepatic and extrahepatic bile ducts in a mouse model of primary sclerosing cholangitis.

**Figure 3:**
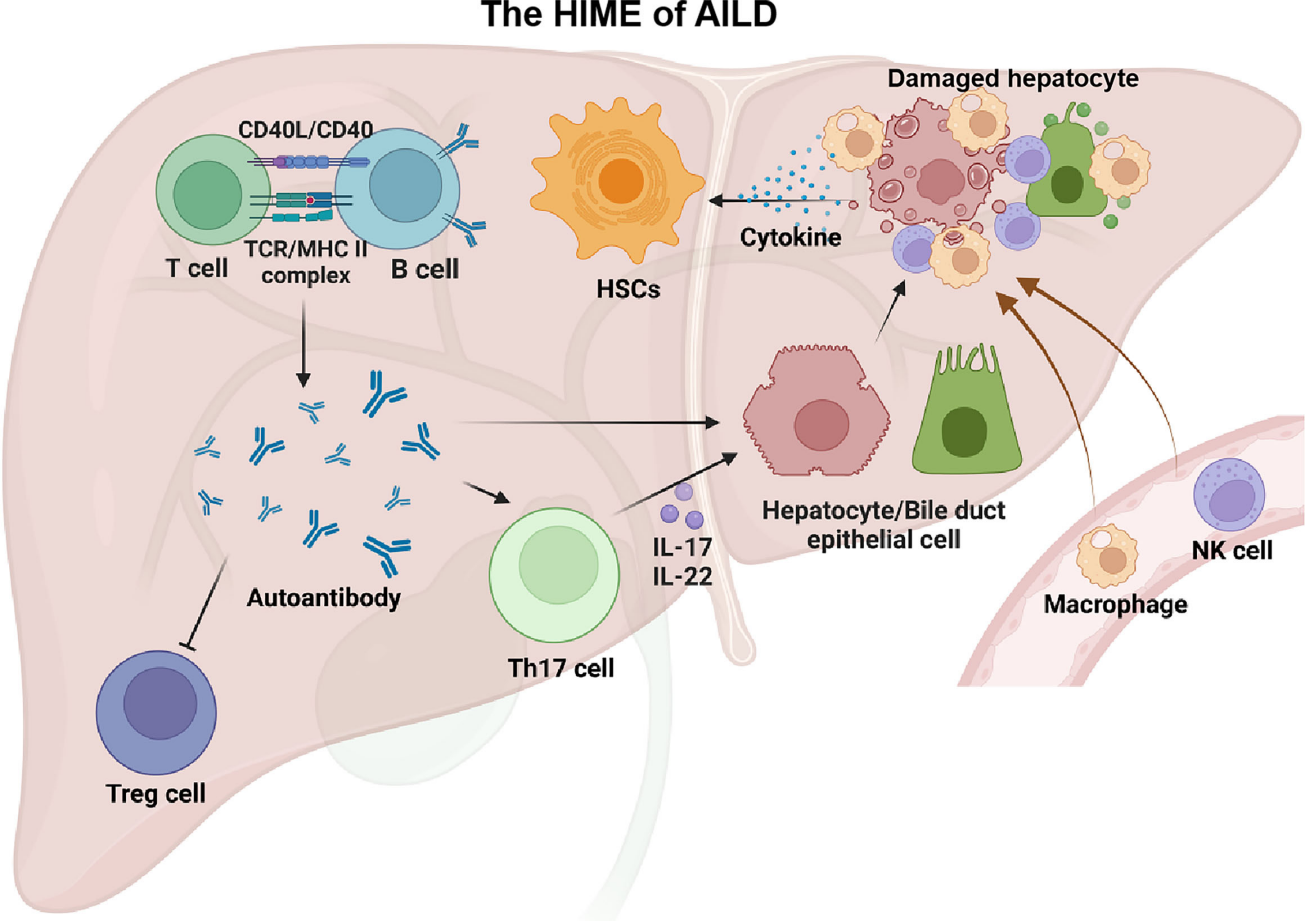
The HIME of AILD. Autoimmune liver disease happens when a large number of autoantigens are present in the liver. With the assistance of T cells, B cells can generate a large number of autoantibodies that recognize these autoantigens. These autoantibodies can impair the function of Treg cells and lead to autoimmune damage. In addition, autoantibodies can directly, or indirectly through Th17 cells, exert an effect on targeted cells (e.g., hepatocytes, biliary epithelial cells) and recruit peripheral macrophages and NK cells, causing liver injury. These injured cells release large amounts of cytokines thereby driving the HSCs activation. Created with BioRender.com.

Regulatory T cells (Tregs)/Th17 cell imbalance is critical for immune disorders in patients with AILD. Tregs are circulating auto-reactive T cells that limit autoimmune damage. A significant decrease in Tregs was found in the peripheral blood and liver of PBC patients (100, 101). In addition, Zhu et al. (102) found that knockout of AMP-activated protein kinase α1 and Tregs-specific deletion could weaken the suppressive activity of Tregs and lead to the development of alcoholic liver disease(ALD) in mice. Th17 cells were significantly increased in the bile ducts of patients with primary biliary cholangitis (103). Th17 cells are helper T cells differentiated from Th0 cells stimulated by IL-6 and IL-23, which can secrete a large number of pro-inflammatory factors such as IL-17 and IL-22, activate TNF or Fas/FasL pathway to mediate hepatocyte apoptosis, and activate HSCs to promote the development of hepatic fibrosis (104, 105). **Figure 3** presented the main alterations of the HIME in the AILD.

### The HIME of fatty liver disease

Fatty liver disease is mainly caused by triglyceride accumulation in the liver leading to continuous hepatocyte stimulation and arousing long-term inflammatory responses. According to whether exposure to long-term excessive alcohol consumption fatty liver disease can be divided into ALD and non-alcoholic fatty liver disease (NAFLD).

***ALD*** As ALD patients often consume amounts of alcohol for a long time, the majority of ethanol is metabolized in the liver. Acetaldehyde, the intermediate product of ethanol, can directly damage hepatocytes and bind with the intracellular proteins as antigens to cause humoral immune responses (106, 107). This tissue stress injury leads to an aseptic inflammation (108), which further activates the KCs, initiating the immune response and recruiting peripheral monocytes into the liver. And the KCs are capable of releasing the number of inflammatory factors and chemokines such as TGF-β, TNF-α, and IL-1β to stimulate HSCs activation. Furthermore, macrophages of the M2 type possess a powerful activating effect on HSCs (109, 110). Ethanol-induced telomerase reverses transcriptase expression in macrophages and promotes macrophage polarized into M1 type through the NF-κB signaling pathway (111). And ethanol can activate HSCs to induce collagen deposition by up-regulating P2X7 receptor (P2X7R) expression-an ATP-gated, non-selective cation channel receptor belongs to the P2X family of P2 purine receptor in human macrophages leading to NLRP3 inflammasome activation and releasing the IL-1β. Inhibition of NLRP3 inflammasome activation effectively suppresses further deterioration of alcoholic steatohepatitis and attenuates alcohol-induced liver injury (112–114). Another study found P2X7R can also mediate acetaldehyde-induced HSC activation *via* the PKC-dependent GSK3β pathway (115). Additionally, ethanol can also directly affect the expression of epigenetic regulators during HSC transdifferentiation. The histone modifying enzymes such as H3K4 and methyltransferase MLL1 are upregulated in HSCs, when exposed to ethanol and recruited to the elastin gene promoter, resulting in the up-regulation of pro-fibrotic elastin genes expression and increased ECM protein expression.

ROS also plays an important role in hepatic fibrosis resulting from alcoholic liver disease. Macrophages exposed to chronic ethanol can induce ROS release, which acts as an inducer of the TGF-β signaling pathway, thereby promoting the activation of HSCs. The dual effects of ethanol and ROS promote hepatocyte lysis and death, releasing the DAMPs to the extracellular matrix, stimulating macrophage activation, and initiating liver regeneration as well as fibrosis processes (116, 117). ATP, as DAMPs released during cell lysis, acts as an endogenous danger signal to activate intracellular inflammatory response through P2X7R and exacerbate cell damage (118).

A recent study found that alcohol intake increases intestinal permeability, promotes the transfer of endotoxins such as lipopolysaccharide (LPS) from Gram-negative bacteria to the portal vein, and reaches the liver with the bloodstream. LPS activation of various TLR ligands was shown to induce miR-155 in macrophages. miR-155 plays a critical role in promoting macrophage M2 polarization. miR-155 also targets and activates SMAD2/5, Snail1, STAT3 genes that are involved in fibrosis. And the miR-155 knockout protects mice from alcohol-induced steatosis and inflammation (119–121).

**Figure 4:**
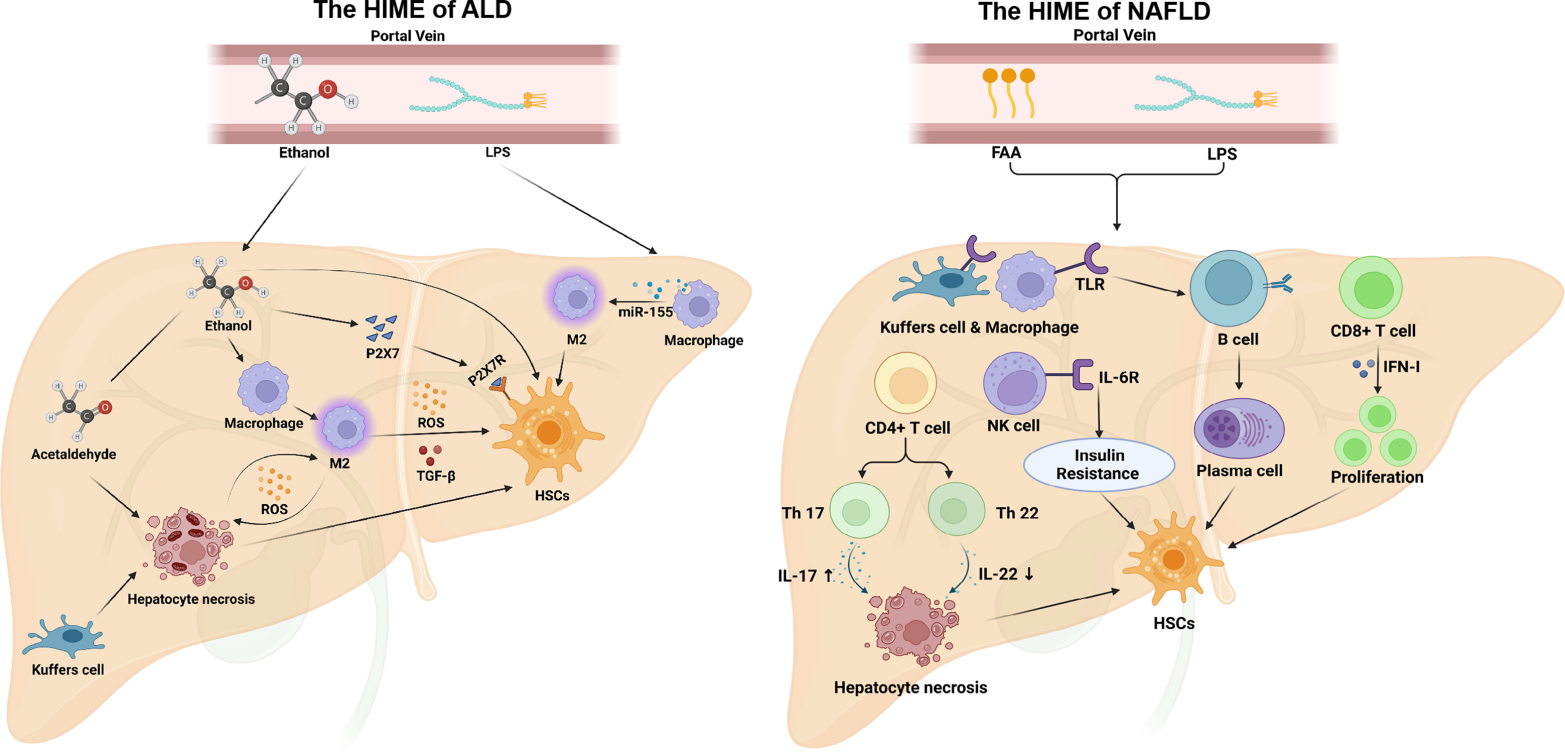
The HIME of ALD and NAFLD. In ALD, ethanol is absorbed through the intestine and entry the portal vein to the liver. Ethanol can stimulate M2 macrophage polarization that secretes reactive oxygen species and TGF-β to promote hepatic fibrosis. Ethanol also increases P2X7R(P2X7 receptor) expression on HSCs, and P2X7 can promote HSCs activation. Acetaldehyde, an intermediate product of ethanol, induces hepatocyte necrosis. And necrotic hepatocytes directly activate HSCs and release ROS to activate M1 macrophages. Additionally, ethanol can disrupt the intestinal barrier, causing LPS to enter the liver *via* the portal vein and induce M2 macrophage polarization. In NAFLD, free fatty acid and LPS from the peripheral circulation induce TLR expression in macrophages, transform B cells into plasma cells, and secrete antibodies, which activate HSCs activation. In addition, free fatty acid and LPS are also able to stimulate CD4^+^ T cells, regulate the balance of Th17/Th22 cells, and induce hepatocyte necrosis by increasing IL-17 and reducing IL-22. Moreover, CD8^+^ T cells and NK cells can also promote HSCs activation under this HIME. Created with BioRender.com.

***NAFLD*** NAFLD is characterized by excessive lipids accumulation in the liver, which can affect bile acid metabolism, hepatocyte metabolism, and the function of macrophages and HSCs. And it can promote the progression of NAFLD to NASH or even cirrhosis by activating the metabolism of intrahepatic immune cells (122)(**Figure 4**). Innate immune activation is a key factor in the exacerbation of hepatic inflammation in NAFLD/NASH. The TLR was reported to mediate B-cell activation through activation of myeloid differentiation primary response protein 88 (MyD88) in a high-fat high-carbohydrate diet-fed mouse model of NASHA. The activated B cells secreting pro-inflammatory cytokines, modulating neighboring immune cells, and differentiating into antibody-secreting cells will result in the progression of NAFLD. A study reported that transplantation of gut microbiota from NAFLD patients into recipient mice can also increase B cell accumulation and activation in the liver (123). Studies have shown that insulin resistance is often accompanied by NAFLD-associated hepatic fibrosis, and NK cells contribute to the development of obesity-associated insulin resistance. Lipid accumulation promotes IL-6R expression in mouse NK cells, and IL-6/STAT3-dependent myeloid NK cell subsets are a critical determinant of NAFLD-associated insulin resistance *in vivo* (124). Almost all CD4^+^ T cells are involved in the sterile inflammation associated with NASH. IL-17 secreted by Th17 cells increases c-Jun N-terminal kinase (JNK) activation in steatotic hepatocytes and exacerbates hepatocyte injury, while IL-22 secreted by Th22 cells protects hepatocytes by inhibiting PI3K/Akt-mediated JNK activation. And the balance breaking between IL-17 and IL-22 will lead to hepatocyte injury and accelerate NAFLD progression (125). Ghazarian et al. (126) reported that high-fat diet (HFD) feeding increased the expression of IFN-αR on CD8^+^ T cells and upregulated the key transcription factors of IFN-I in the liver of the NAFLD mouse model. While the activation of IFN-I responses drives the expansion of intrahepatic pathogenic CD8^+^ T cells, leading to the NAFLD progression. In mouse models of NAFLD, saturated free fatty acid (FFA) levels are increased in the blood, and they can bind to TLR2 and TLR4 of liver-resident Kupffer cells and peripherally infiltrating macrophages, triggering multiple mechanisms to produce the ROS, such as mitochondrial damage, endoplasmic reticulum stress, and nicotinamide adenine dinucleotide phosphate oxidase. Nicotinamide adenine dinucleotide phosphate oxidase2-derived ROS could stimulate macrophages to produce pro-inflammatory cytokines, such as TNF-α, IL-6, and IL-1β, which promote the development of hepatic fibrosis by up-regulating NF-κB pathway and activate JNK activator protein-1 in HSCs (127). Another study reported a high-fat diet-induced increased bile acid secretion, resulting in gut microbiota disturbances and intestinal barrier dysfunction in a mouse model of NAFLD (128). It will lead to an increase of LPS and other bacteria-derived compounds in portal blood, promoting the activation of TLR and other pattern recognition receptors in the liver, and triggering local inflammatory and fibrotic responses. In addition, in a mouse model of induced NASH-fed diets rich in palmitate, cholesterol, and sucrose, the palmitate of hepatocyte specifically promotes the overexpression of the Notch pathway ligand Jag1 by activating TLR4 to upregulate NF-κB signaling. And the activation of the Notch signaling pathway in hepatocytes drives the expression of genes encoding osteopontin and secreted phosphoprotein 1 (Spp1), which promotes HSCs activation and hepatic fibrosis (129).

## Effects of regulating the HIME for preventing hepatic fibrosis

The importance of immune cell populations in the development of chronic liver disease is self-evident. However, most traditional treatments for liver fibrosis are still focused on inhibiting the activation of HSCs. The role of HIME in liver fibrosis should not be underestimated. Therefore, blocking the infiltration of immune cells in the blood or altering the function of infiltrating immune cells or cytokines may be a potential target for the treatment of liver fibrosis. **Table 1** summarized the details of the effects of traditional Chinese medicine(TCM) in modulating the HIME for preventing hepatic fibrosis of chronic liver disease. In four preclinical studies, researchers regulated the functions of HSCs (130, 132), macrophages or KCs (25, 131, 134–136, 138), regulatory T cells (133), DCs (30), and NK cells (25, 137) by different treatment approach in the CCl4 or bile duct ligation -induced liver fibrosis murine model. And these interventions can effectively alleviate hepatic fibrosis and tissue inflammation. Another study found inhibiting the chemokines CCR2/5 could significantly reduce circulating Ly6C^hi^ monocytes and hepatic monocyte-derived macrophage infiltration in murine models of NASH, and attenuate hepatic inflammation and fibrosis (140). A chemical agent, dopamine receptor D2 antagonism, was screened to target the Yes-associated protein signaling pathway in macrophages. It antagonizes Yes-associated protein-dependent fibrotic crosstalk between selectively targeted macrophages and CTGF + VCAM1 + vascular niche, blocks fibrosis, and restores liver architecture in rodent and large animal models of NASH (139).

**Table 1:** The effects of regulating the HIME on the progression of hepatic fibrosis.

Author, publishing year [ref]	Species and disease models	Therapeutic methods	Targeted cells; Signaling pathway	The mechanisms of therapeutic effect	Effects on progression of hepatic fibrosis
Xiafei Wu, 2015 (130)	CCl4-induced hepatic fibrosis rat model	Tetramethylpyrazine (TMP)	HSCs; PDGF-bR/NLRP3/caspase1 pathway	TMP suppressed inflammation by decreasing levels of inflammatory cytokines, including TNF-a, NLRP3, NF-kB and IL-1b, and significantly protected the liver from injury and fibrogenesis; TMP promotes the role of PDGF-bR/NLRP3/caspase1 pathway; TMP improved histological structure of liver and decreased hepatic enzyme levels and collagen deposition in fibrotic liver.	TMP reduced hepatic inflammation and fibrosis
Pengfei Ma, 2017 (25)	CCl4 or BDL-induced hepatic fibrosis murine model	Bone-marrow-derived macrophages were polarized into M0, M1, or M2 macrophages	Macrophages and NK cells	M1 Macrophage produced matrix metalloproteinases and hepatic growth factor, which promoting collagen degradation and hepatocyte proliferation; increasing the activated NK cells in fibrotic liver and inducing HSCs apoptosis.	Bone-marrow-derived M1 macrophages alleviated hepatic fibrosis
Ying Xu, 2018 (131)	CCl4-induced hepatic cirrhosis rat model	Yiguanjian (YGJ)	Fetal liver stem/progenitor cells (FLSPC) and macrophages; Wnt/beta-catenin pathway	1.YGJ suppressed the macrophages activation and inhibited non-canonical Wnt and promoted canonical Wnt signaling pathway; 2. YGJ promoted the liver regeneration differentiation of FLSPCs into hepatocytes.	YGJ enhanced FLSPC-mediated repair of hepatic cirrhosis
Fang Gan, 2018 (132)	CCl4-induced hepatic fibrosis rat model	Lycium barbarum polysaccharides (LBPs)	HSCs; TLRs/NF-kB pathway	LBPs decreased α-smooth muscle actin expression and the activity of aspartate transaminase, alkaline phosphatase and alanine aminotransferase; LBPs alleviated CCl4-induced oxidative injury, inflammatory response and TLRs/NF-kB signaling pathway expression.	LBPs alleviated hepatic fibrosis
Yejin Xu, 2019 (133)	CCl4-induced hepatic fibrosis murine model	IL-10 Gene-modified bone marrow-derived dendritic cells therapy	Regulatory T Cells; TGF-β/Smad pathway	IL-10 Gene-modified bone marrow-derived dendritic cells therapy increased regulatory T cells, while ALT, AST and inflammatory cytokines were significantly reduced, and the TGF-β/smad pathway was inhibited.	The dendritic cells therapy alleviated hepatic fibrosis
Zhi Ma, 2022 (134)	BDL-induced hepatic fibrosis murine model	Si-Wu-Tang (SWT) decoction	Macrophages, neutrophils, CD8+ T cells and HSCs; Fas/FasL pathway	SWT inhibited the activation of M2 macrophages to reduce the release of profibrotic-cytokines and prevented the activation of neutrophils; SWT increased the CD8^+^ T cells and promoted activated HSCs apoptosis through Fas/FasL pathway.	SWT improved hepatic fibrosis and inflammatory responses
Ming Xiang, 2022 (30)	CCl4 -induced hepatic fibrosis murine model	Kinsenoside (KD)	DC cells and CD8^+^ T cells; PI3K-AKT-FoxO1 pathway	KD inhibited DCs maturation; KD promoted PD-L1 expression via PI3K-AKT-FoxO1 pathway; KD reduced IL-12 expression, blocked activation of CD8^+^ T cells and HSCs, and reduced α-smooth muscle actin and Col-I expression.	KD alleviated hepatic fibrosis
Peng Liu, 2022 (135)	CCl4 -induced hepatic fibrosis murine model	Chitooligosaccharide (COS)	Kuffer cells and macrophages; JAK2/STAT1 pathway and JAK1/STAT6 pathway	COS inhibited polarization of M1 and M2 macrophages; COS inhibited JAK2/STAT1 pathway on M1 macrophages and JAK1/STAT6 pathway on M2 macrophages; 3. COS rescued mice from hepatic fibrosis, marked by decreased deposition of extracellular matrix and histological lesions.	COS alleviated hepatic fibrosis
Xiang-an Zhao, 2022 (136)	CCl4 -induced hepatic fibrosis murine model	Curcumin	Kupffer cells and Ly6C(hi) MoMFs; ERK1/2 and p38 pathway	1. Curcumin decreased intrahepatic Ly6C(hi) monocyte infiltration as well as associated pro-inflammatory and profibrotic cytokines; 2. Curcumin reduced KCs activation and monocyte chemokines; 3. Curcumin prevented the M1 polarization of macrophages through ERK1/2 and p38 pathway.	Curcumin protect against hepatic fibrosis
Xixi Tao, 2022 (137)	CCl4 or BDL -induced hepatic fibrosis murine model	Sulprostone	NK cells and HSCs	Deletion of EP3 impaired the cytotoxicity of NK cells toward HSCs; EP3 upregulated Itga4 expression in NK cells through promoting Spic nuclear translocation.	Activation of EP3 by sulprostone alleviated hepatic fibrosis
Jianhua Rao, 2022 (138)	CCl4 or BDL or MCD diet induced hepatic fibrosis murine model	Myeloid-specific Follistatin-like protein 1 (FSTL1)-knockout; Activation of pyruvate kinase M2	Macrophages; TLR-4/NF-κB pathway	FSTL1 deletion results in diminished hepatic macrophage and neutrophil infiltration and reduced expression of pro-inflammatory factors FSTL1 deletion inhibits M1 polarization and TLR-4/NF-κB pathway activation in macrophages; Pyruvate kinase M2 was able to counteract FSTL1-mediated M1 polarization in macrophages and inflammation	Myeloid-specific FSTL1 deficiency alleviated hepatic fibrosis
Jie Qing, 2021 (139)	CCl4-induced NASH murine and minipig models; liver biopsies from patients with cirrhosis	Dopamine receptor D2 antagonism (DRD2)	Macrophage; Hippo/YAP pathway	1. DRD2 antagonizes YAP-dependent fibrotic crosstalk promoting liver regeneration over fibrosis; 2. DRD2 antagonists block fibrosis and restore liver architecture in rodent and large animal models of NASH.	DRD2 antagonists block fibrosis and restore liver architecture in NASH
Tobias Puengel, 2022 (140)	Serum samples and liver biopsies from patients with NAFLD; CDAHFD induced NASH murine model	Chemokine receptor 2/5 (CCR2/5) inhibitor and pegylated fibroblast growth factor 21 (FGF21) agonist	Ly6C(hi) MoMFs	In NAFLD patients, serum levels of chemokines CCL2 was associated with inflammation and advanced hepatic fibrosis, FGF21 was associated with inflammation; In murine models of NASH, CCR2/5 inhibition reduced circulating Ly6C(hi) MoMFs, and the FGF21 agonist reduced body weight, hepatic triglycerides.	CCR2/5 inhibition and FGF21 agonism ameliorates steatohepatitis and hepatic fibrosis

CCl4, carbon tetrachloride 4; HSCs, hepatic stellate cells; TNF, tumor necrosis factor; NF-kB, nuclear factor kappa B; IL, interleukin; BDL, bile duct ligation; NK cells, natural killer cell; DC cells, Dendritic Cells; NASH, nonalcoholic steatohepatitis; NAFLD, non-alcoholic fatty liver disease; CDAHFD, choline-deficient, L-amino acid-defined, high-fat diet; Ly6C(hi) MoMFs, Lymphocyte antigen 6C (high) monocyte-derived macrophages.

PDGF-bR/NLRP3/caspase1 pathway: platelet-derived growth factor-b receptor/NOD-like receptor thermal protein domain associated protein 3/caspase1 pathway.

TLRs/NF-kB pathway: Toll-like receptors/ nuclear factor kappa B pathway.

TGF-β/Smad pathway: transforming growth factor β/Smad pathway.

PI3K-AKT-FoxO1 pathway: phosphatidylinositol 3-kinase/protein kinase B/forkhead box O1 pathway.

JAK/STAT pathway: Janus tyrosine kinase/signal transducer and activator of transcription pathway.

ERK1/2 and p38 pathway: extracellular signal regulated kinase 1/2 and p38 signaling pathway.

The occurrence and progression of liver fibrosis often involve a variety of cells and multiple signaling pathways. Clinical TCM treatments are advanced in action on multi-cellular and multi-target, which might be a new choice for alleviating liver fibrosis. Ginsenoside (KD), the active ingredient in the TCM ginseng, was reported could reduce hepatic histopathological damage, proinflammatory cytokine release, and extracellular matrix deposition in CCl4-induced hepatic fibrosis. The KD restrained the hepatic fibrosis-driven rise in CD86, MHC-II, and CCR7 levels, while upregulated PD-L1 expression on DCs, which blocked CD8^+^ T cell activation. Additionally, KD reduced DC glycolysis, maintained DCs immature, and was accompanied by an IL-12 decrease in DCs. These effects disturbed the communication of DCs and HSCs with the expression or secretion of α-smooth muscle actin and Col-I declined in the liver (30). Lycium barbarum polysaccharides (LBPs) supplementation was reported to reduce CCl4-induced oxidative injury, inflammatory response, and TLRs/NF-kB signaling pathway expression, which alleviated CCl4-induced liver fibrosis (132). Moreover, two Chinese herbal formulas Si-Wu-Tang (134)and Yi Guan Jian (131) were reported to regulate the activation and polarization of macrophage, which significantly improved liver fibrosis and inflammatory responses. Additionally, Si-Wu-Tang also can inhibit the M1 from inhibiting the infiltration of neutrophils and promote the CD8^+^ organization to reside memory T cells cytotoxic effect inducing the HSCs apoptosis (134). The Yi guan Jian can enhance the therapeutic effects of fetal liver stem/progenitor cells that promoted the liver regeneration differentiation of fetal liver stem/progenitor cells into hepatocytes (131). Collectively the data supports the notion that multi-target therapeutics may be an effective option for t liver fibrosis treatment (**Table 1**).

## Concluding and prospects

The immune cells and HSCs could synthesize and secrete several chemical mediators under chronic liver injury, and they constitute a dynamic HIME of an immune cells-cytokines-chemokines network system that affects the processes of hepatic fibrosis. It can be seen the HIME of hepatic fibrosis is complex not only because there are a variety of cells involved, but also because there are different sub-types of immune cells, which play different roles at different stages of liver disease progression. Moreover, in addition to local immune cells in the liver, a variety of immune cells in the circulation also can be chemotactic into the liver tissue and differentiate into different cell subtypes, exerting an effect on the initiation, progression, and regression of hepatic fibrosis. According to the summary and analysis in this review, most sub-types of immune cells have bidirectional effects on the development of hepatic fibrosis, because the immune cells interact with each other and also with other stromal cells in the microenvironment through diverse and complex cytokines networks, and eventually achieve a balance. While this balance will be broken with the persistence or elimination of the disease’s causes, then these cells will form a new balance through the other signaling pathways and exert a promoting or inhibiting role in hepatic fibrosis to a different degree. Therefore, it is necessary not only to explore the function of a certain group of immune cells, but also to explore cells’ diversity, and accurately analyze the cells’ specific phenotype and role in different liver diseases, different stages of the same disease, and different HIME caused by different pathogenic factors stimulus. Recently, single-cell sequencing can clarify the specific role of single cells. If this detection method is applied to the investigation of the immune microenvironment of hepatic fibrosis, it may greatly enrich the existing immune network and provide comprehensive and accurate ideas for the study of hepatic fibrosis. Additionally, the specific mechanisms for the immune cells of a specific phenotype to play different roles at different stages of chronic liver disease need to be elucidated, so that a precise intervention can be performed. However, the study on specific mechanisms is currently a major vacancy for the effect of HIME on hepatic fibrosis, thus, future studies need to focus on a deeper and more specific regulatory pathway to achieve an accurate intervention target, thereby delaying or avoiding the occurrence and progression of hepatic fibrosis.

## Author contributions

NZ, HY, and ZZ conceived of the subject matter and wrote the logic of this review, searched, categorized, and summarized all relevant literature, and wrote the initial draft. ZL, XC, YZ, and RJ helped to check relevant information check the content of the initial draft. JH and HP edited the second draft and composed the manuscript. XL and YL reviewed and edited the manuscript. All authors contributed to the article and approved the submitted version.
